# A Study of the Identification, Fragmentation Mode and Metabolic Pathways of Imatinib in Rats Using UHPLC-Q-TOF-MS/MS

**DOI:** 10.1155/2021/8434204

**Published:** 2021-05-24

**Authors:** Sijiang Liu, Zhaojin Yu

**Affiliations:** ^1^Department of Pharmaceutical Sciences, China Medical University-The Queen's University of Belfast Joint College, China Medical University, 77 Puhe Road, Shenyang 110122, China; ^2^Department of Pharmacology, School of Pharmacy, China Medical University, Shenyang 110122, China; ^3^Liaoning Key Laboratory of Molecular Targeted Anti-Tumor Drug Development and Evaluation, Liaoning Cancer Immune Peptide Drug Engineering Technology Research Center, Key Laboratory of Precision Diagnosis and Treatment of Gastrointestinal Tumors (China Medical University), Ministry of Education, China Medical University, Shenyang 110122, China

## Abstract

In this study, The metabolites, metabolic pathways, and metabolic fragmentation mode of a tyrosine kinase inhibitor- (TKI-) imatinib in rats were investigated. The samples for analysis were pretreated via solid-phase extraction, and the metabolism of imatinib in rats was studied using ultra-high-performance liquid chromatography-quadrupole-time-of-flight mass spectrometry (UHPLC-Q-TOF-MS/MS). Eighteen imatinib metabolites were identified in rat plasma, 21 in bile, 18 in urine, and 12 in feces. Twenty-seven of the above compounds were confirmed as metabolites of imatinib and 9 of them were newly discovered for the first time. Oxidation, hydroxylation, dealkylation, and catalytic dehydrogenation are the main metabolic pathways in phase I. For phase II, the main metabolic pathways were N-acetylation, methylation, cysteine, and glucuronidation binding. The fragment ions of imatinib and its metabolites were confirmed to be produced by the cleavage of the C-N bond at the amide bond. The newly discovered metabolite of imatinib was identified by UHPLC-Q-TOF-MS/MS. The metabolic pathway of imatinib and its fragmentation pattern were summarized. These results could be helpful to study the safety of imatinib for clinical use.

## 1. Introduction

**Figure 1:**
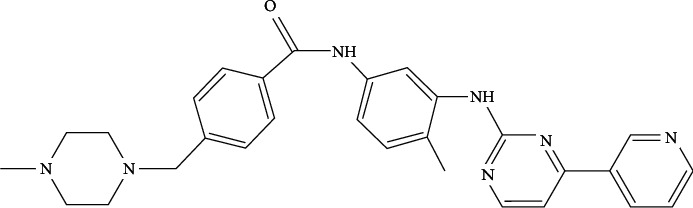
The structure of imatinib.

Imatinib (IM) is the first generation of tyrosine kinase inhibitor [1, 2] mainly used for the treatment of chronic, accelerated phase or blast crisis of adult chronic myeloid leukemia with positive Philadelphia chromosome and malignant gastrointestinal stromal tumors that cannot be resected and/or metastasized [3, 4]. IM was first tested in clinical trials in the 1990s and was approved by the US Food and Drug Administration (FDA) at the beginning of this century. Its antitumor mechanism specifically blocks the binding site of adenosine triphosphate (ATP) on tyrosine kinase to inhibit the autophosphorylation and substrate phosphorylation of BCR-ABL target protein. IM could also stabilize the inactive conformation of tyrosine kinase to inhibit its activity, leading to reduced proliferation of chronic myelocytic leukemia (CML) cells and cell apoptosis [5–7]. IM has excellent pharmacokinetic characteristics and its oral bioavailability was reported to be ≥ 98%, with a half-life of 20 hours and peak time ranging from 2 to 4 hours. Its excretion pathway mainly depends on feces and urine [8]. The chemical name of IM is 4-[(4-methyl-1-piperazinyl) methyl]-N-[4-methyl-3-[4-(3-pyridyl)-2-pyrimidinyl] amino] phenyl] benzamide, and its chemical structure is shown in Figure 1.

Most drugs were mainly eliminated from the body after metabolism [9–11]. Metabolism can be understood as the process of converting drugs into their metabolites under the catalysis of enzymes [12]. Drug metabolism may produce one or more active metabolites with the same pharmacological effects and targets as the original drug [13, 14]. Sometimes, it may also generate overactive or toxic metabolites, which may cause serious adverse side-effects [15]. For example, some CML patients developed hepatotoxicity symptoms after using IM, which may be related to one or more metabolites of IM [16]. In addition, studies showed that the main metabolites of IM may be involved in IM resistance [17]. Therefore, to systematically understand the safety of IM for its clinical application, it is necessary to investigate its metabolites. Up to date, researchers have done a lot of work on IM metabolism and achieved reliable results. MarcMarull et al., with the aid of liquid chromatography combined with triple quadrupole mass spectrometer (TSQ-MS) and linear ion trap mass spectrometer (LTQ-MS), successfully isolated one demethylated metabolite, two hydroxylated metabolites, and three N-oxidation metabolites of IM from microsomes containing cytochrome P450 (CYP) isozymes [18]. Li et al. found 7 cyano metabolites of IM from human liver microsomes (in vitro) and identified their structures [19]. Friedecký et al. have identified 90 metabolites of IM from the plasma of patients with CML [20]. Therefore, a thorough study of the metabolites and metabolic pathways of imatinib will help to use IM safely and rationally. In addition, the study of drug metabolites of IM will help with its clinical efficacy and provide more information for the scientist to develop safer and more effective formulations than the raw materials [21, 22].

The purpose of this study was to investigate the metabolism of IM in rats. The metabolites of IM in plasma, bile, urine, and feces were identified using UHPLC-Q-TOF-MS/MS. The metabolic pathways of phases I and II and the fragmentation pattern of metabolites were also summarized.

## 2. Materials and Methods

### 2.1. Chemicals and Reagents

Imatinib (purity, ≥99.0%) was obtained from Shanghai Macklin Biochemical Technology Co., Ltd., China. HPLC grade acetonitrile and formic acid were purchased from Fisher Scientific (Fair Lawn, NJ, USA). Methanol (analytical or HPLC grade) was procured from Yuwang Industrial Co., Ltd. (Shandong, China). Purified water was provided by Wahaha Group Co., Ltd. (Hangzhou, China).

### 2.2. Animal Experiments

Male Sprague-Dawley (SD) rats (*n* = 18, weighing 220–250 g, 12–14 weeks old) were provided by the Experimental Animal Center of Shenyang Pharmaceutical University (Shenyang, China). The SD rats were fed in metabolic cages for at least 3 days and fasted for 12 h before the experiments. All experiments *in vivo* were conducted under the guidelines of the administration of experimental animals in China.

IM (50 mg/mL) was dissolved in 0.5% carboxymethyl cellulose sodium (CMC-Na) aqueous solution. After administering IM solution at a dose of 50 mg/kg, the rats were randomly divided into three groups (six rats in each group).

Group I was for taking the plasma samples. The blood was taken from the ophthalmic veins at time points at 0.5, 1, 2, 4, 8, and 12 h after oral administration of IM, and then the blood collected in heparinized tubes was centrifuged immediately at 5000 rpm for 10 min to obtain the plasma. For Group II (bile samples), the rats were anesthetized with urethane solution (0.05 mL/10 g). Duct cannulation operation was performed to collect blank bile first, and the rats were allowed to recover from anesthesia before drug administration. The samples were continuously collected but samples were divided according to the period of 0–4, 4–8, 8–12, 12–24, and 24–48 h. Group III was used for obtaining the urine and feces samples. The rats were kept in metabolic cages individually and orally given IM, and then, the samples were collected at different time intervals of 0–4, 4–8, 8–12, 12–24, and 24–48 h. All the blank samples of each group were collected before oral administration of IM. All collected samples were stored at −80°C until further treatment.

### 2.3. Sample Pretreatment

Pretreatment of plasma: 200 *μ*L of plasma sample was extracted using 600 uL of methanol and then vortexed for 1 min to precipitate the proteins. After centrifugation at 12000 rpm for 10 min at 4°C, the supernatant was transferred into a clean tube and dried under nitrogen gas at 40°C.

Pretreatment of feces: feces sample (0.2 mg) was added to methanol (2 mL), then vortexed for 2 min, followed by sonication for 30 min, and vortexed again for another 2 min. After the mixture was centrifuged (13000 rpm, 10 min) at 4°C, the supernatant was collected and dried with nitrogen flow at 40°C.

Pretreatment of urine and bile: the solid-phase extraction cartridges (Angela Bond Elut C-18, 1 g) were activated via duplicated wash using water (1 mL), followed by methanol (1 mL). After centrifugation (13000 rpm) for 5 min, the supernatant was filtrated through a 0.22 *μ*m membrane filter, followed by further purification (1 mL of the filtered sample) with a solid-phase extraction cartridge at a flow rate of 30 drops ·min^−1^. The cartridges were washed with water (1 mL) first and then washed for another 3 times using 1 mL of 0.1% formic acid in methanol. The eluent of 0.1% formic acid in methanol was collected and dried with nitrogen flow at 40°C.

Before analysis, all the pretreatment samples have been dried with nitrogen, and the residues were reconstituted with 200 *μ*L of a mixed solvent of acetonitrile and 0.1% formic acid in water in a ratio of 10 : 90 (v/v) and then sonicated for 10 min before centrifugation (12000 rpm) at 4°C. 3 *μ*L of the supernatant was finally injected for UHPLC-Q-TOF-MS/MS analysis.

### 2.4. Instruments and Conditions

The chromatographic separation was carried out using a ACQUITY UHPLCTM BEH C18 column (100 × 2.1 mm, 1.7 *µ*m). Mobile phase A was an aqueous buffer containing 0.1% formic acid and 10 mmol/L ammonium acetate and B was 0.1% formic acid in acetonitrile. The gradient elution program was optimized listed as follows: 10–25% B from 0 to 9.5 min, 25–35% B from 9.5 to 12.5 min, 35–90% B from 12.5 to 20.0 min, and 90–10% B from 22.0 to 22.1 min. Flow rate was 0.3 mL/min; temperature, 40°C; injection volume, 3 *μ*L.

Mass spectrometry detection was performed using an AB SCIEX X500R UPLC-Q/TOF-MS (AB SCIEX, Framingham, MA, USA). Mass spectrum acquisition was in positive electrospray ionization mode and the conditions used were as follows: the pressure of the nebulizer gas (gas 1), the heater gas (gas 2), and the curtain gas was set to 55, 55, and 35 psi, respectively; ion source temperature was 600°C. TOF-MS/MS parameter settings are as follows: scanning range, *m/z* 50∼1000; collision gas, 7 psi; ion spray voltage, 5.5 kV; declustering potential (DP), 80 V; the collision energy (CE), 10 V; TOF-MS/MS, *m/z* 50–1000; ion spray voltage, 5.5 kV; declustering potential (DP), 80 V; the collision energy (CE), from 20 to 50 V; accumulation time, 25 min. Apply AB SCIEX OS for processing. In the experiment, the CDS automatic calibration system was used to calibrate the experimental data collection.

## 3. Results and Discussion

**Figure 2:**
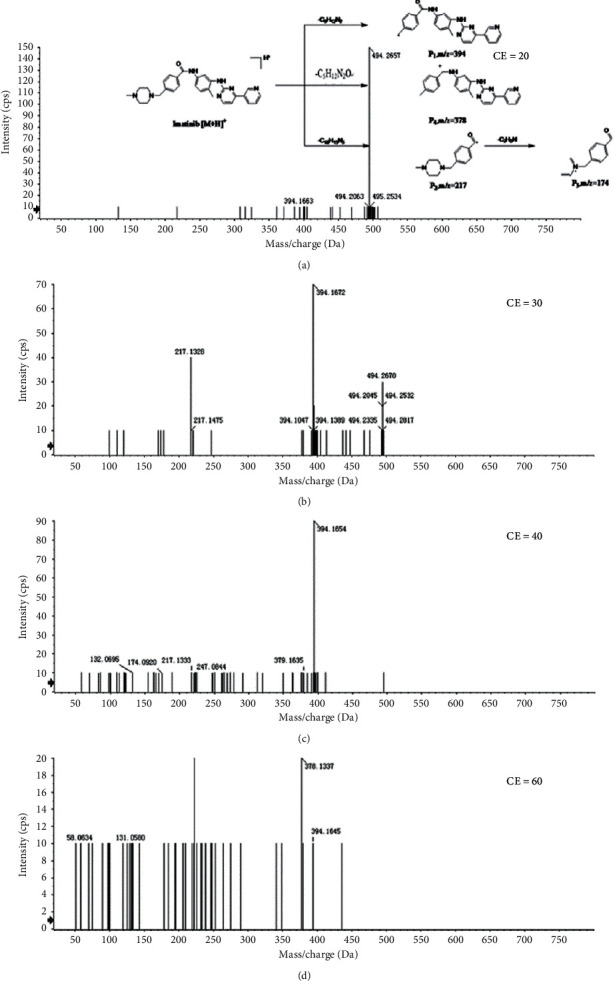
In ESI positive ion mode, different energy collision spectra of [M + H]^+^ of IM and proposed fragmentation pattern of IM.

**Figure 3:**
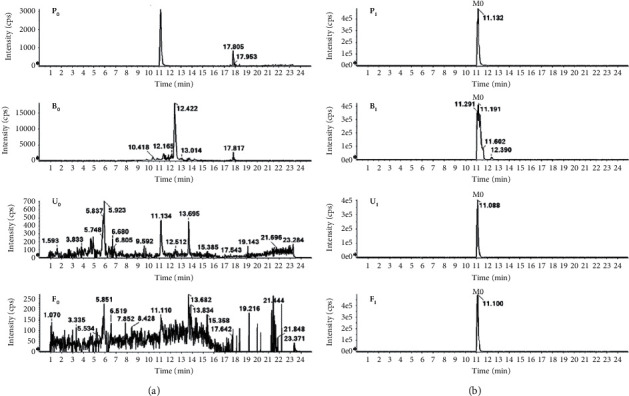
Base peak ion chromatograms of biological samples in positive ion mode. P_0_, B_0_, U_0_, and F_0_ represent blank samples of plasma, bile, urine, and feces from rats; P_1_, B_1_, U_1_, and F_1_ represent plasma, bile, urine, and feces samples from rats after oral administration of IM.

The MS/MS spectrum of IM standard is shown in Figure 2; the ion at *m/z* 394 was formed after the cleavage of the 4-methyl-1-piperazinyl group on benzamide on the phenyl group of 2-aniline pyrimidine, and the ion at *m/z* 394 generated *m/z* 378 by the loss of O of the benzoylamino group. The ion at *m/z* 217 was formed after the cleavage of the amide part of benzamide, and the ion at *m/z* 217 generated *m/z* 174 by RDA (Reverse-Diels-Alder reaction) cleavage reaction. As shown in Figure 3, the analytes eluted at 11.17 min, with the molecular ion [M + H]^+^ of *m/z* 494.2648, were consistent with the ion at *m/z* 394, 378, 217, and 174 of the IM reference standard, indicating that IM (M0) was detected in plasma, bile, urine, and feces of rat. M3, M9, M11, M17, M20, M22-23, and M26-27 were newly discovered metabolites in this study.

### 3.1. Metabolite Analysis in Plasma

**Figure 4:**
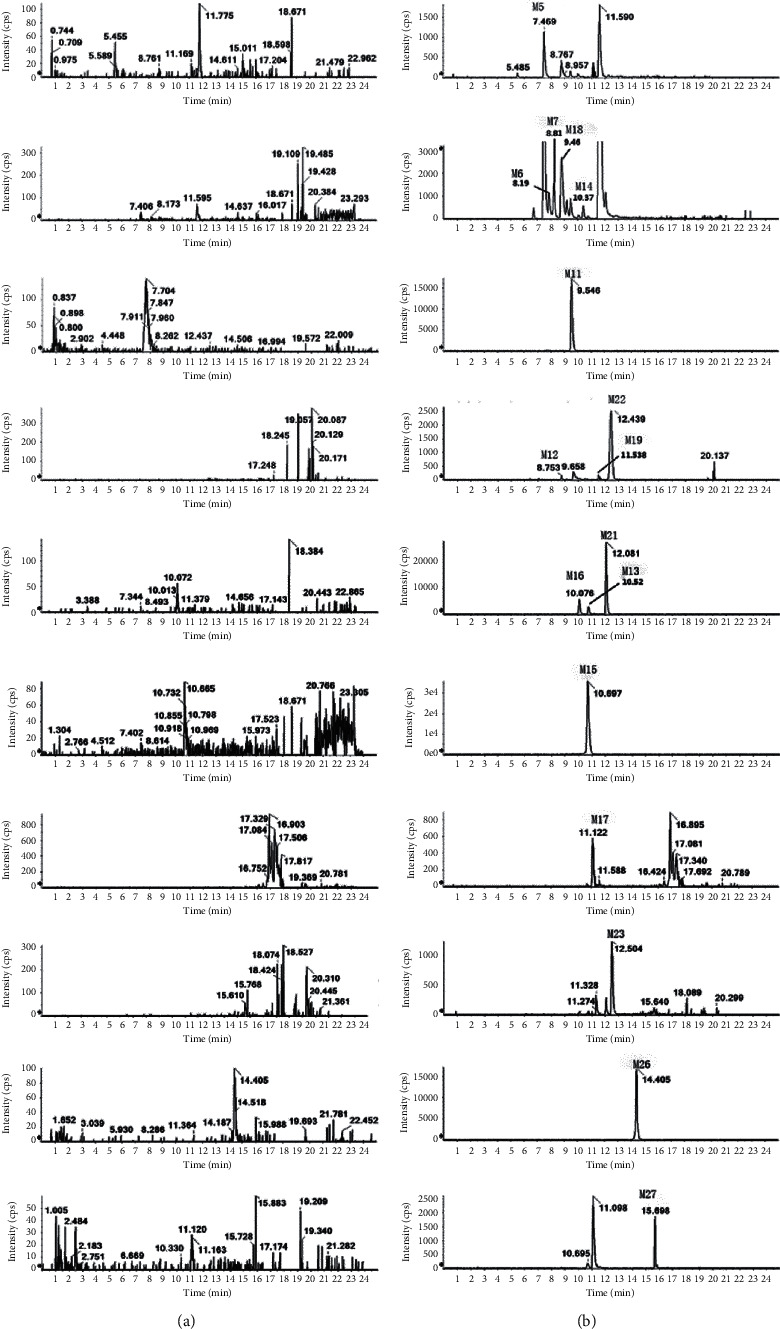
Extracted ion chromatograms (XIC) for metabolites in rats: (a) in blank plasma sample; (b) in plasma sample after an oral administration of IM.

**Figure 5:**
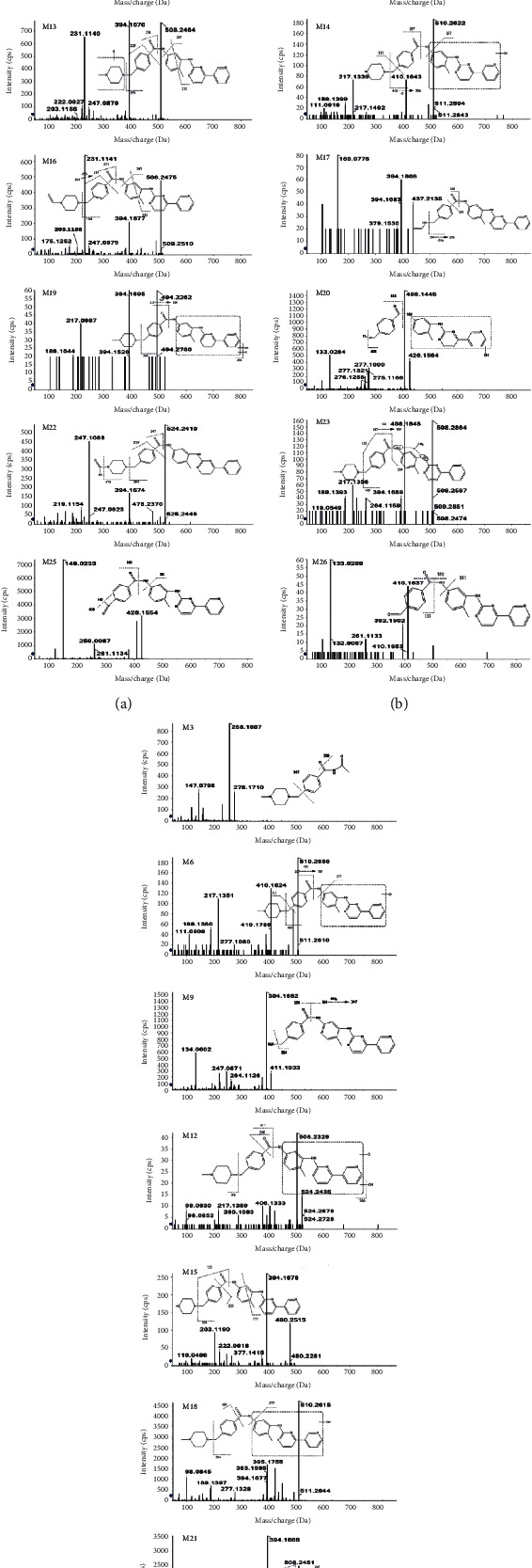
UHPLC-Q-TOF-MS/MS of all metabolites.

Compared with blank samples, there were 6 newly identified metabolites (M11, M17, M22-M23, and M26-M27) in the plasma together with twelve previously reported ones (M5-M7, M10, M12 -M16, M18-M19, and M21). The extracted ion chromatograms of all plasma metabolites are shown in Figure 4. The retention times, excimer ions, and secondary fragment ions (see Table 1) were analyzed together, and the metabolite structures were inferred based on the metabolic patterns of the IM (Figure 5). Moreover, if the structure of some metabolites is different from IM, the structural fracture mode of other known metabolites is used for analysis.

Metabolites of M6, M7, M10, M14, and M18 (C_29_H_32_N_7_O_2_) were detected in MS/MS spectra at 8.19 min, 11.54 min, 10.37 min, 9.46 min, and 8.81 min, respectively. These 5 metabolites were 16 Da (-OH) higher than IM. They had similar MS/MS spectra and produced protonated molecule ions [M + H]^+^ at *m/z* 510.2611, *m/z* 510.2598, *m/z* 510.2610, *m/z* 510.2611, and *m/z* 510.2610. The ions at *m/z* 410 were generated via the broken chemical bond between the methyl groups on the C_4_ position of the benzene ring that were substituted by hydroxyl groups and piperazine group. The remaining fragment ions are related to the fragmentation of the benzamide group. The presence of *m/z* 277 resulted from the fracture of the benzamide group N and hydroxyl substituted 4-methyl-3-[4-(3-pyridyl)-2-pyrimidine. After the amide bond was disrupted, the ion at *m/z* 217 was detected in MS/MS spectra. The loss of C = O (*m/z* 217) results in the emergence of *m/z* 189; then, *m/z* 111 and *m/z* 410 are two fragments produced by a fracture occurring in the same location, and the comprehensive analysis indicates that M6 is a hydroxyl metabolite of IM (substituted at 4-methyl-3-[4-(3-pyridine)-2-pyrimidine). However, the MS^2^ spectra of M7 were observed with one more fragment ion (*m/z* 394) than M6 (*m/z* 410) due to loss of O atom at N-oxidized metabolite. It was speculated that M7 may be the N-oxidation of 4-methyl-3-[4-(3-pyridine)-2-pyrimidine in the IM structure. Compared to M6, M10, and M14, it almost went through the same fragmentation, but the *m/z* 277 fragment ion was not observed, suggesting that there was more N atom substitution on the benzamide group at the hydroxyl substitution position. Therefore, M10 and M14 may be N-oxidized or hydroxyl substituted metabolites of IM (substitution occurs on the N-4-methyl-3-[4-(3-pyridine)-2-pyrimidine structure). The *m/z* 380 fragment ion of M18 was the product of deamination of CH_2_ from one molecule of benzamide by the *m/z* 394 fragment ions, and the other fragment ions were degraded in the same manner as M6. It is presumed that M18 may be an N-oxidized metabolite of IM (occurring on the structure of 4-methyl-3-[4-(3-pyridine)-2-pyrimidine). Among the remaining metabolites, five of them (M13, M15, M16, M21, and M23) had molecular weights that differed from IM by 14 Da. Metabolites M13 and M21 (C_29_H_30_N_7_O_2_) were observed in MS/MS spectra at 10.08 min and 12.06 min; then, they created protonated molecule ions [M + H]^+^ at *m/z* 508.2447 and *m/z* 508.2443, respectively; they were all 14 Da higher than IM. After the C_4_ methyl on the benzene ring of 2-anilinopyrimidine breaks, the site forms a five-membered ring with the C_1_-N of the pyrimidine ring. Then, the chemical bonds between C_1_ and N breaks, *m/z* 247, and fragment ions are generated. The fragment ion at *m/z* 222 was generated by the fracture of C_1_ and C_6_, C_4_, and C_5_ in the fragment ion structure of *m/z* 247 and simultaneously intramolecular proton migration rearrangement. The fragment ion at *m/z* 231 was generated after the amide bond fracture of carbonyl-substituted IM, and the ionic fracture mode of other fragments was the same as that of M6. M13 and M21 are the IM metabolites of the carbonylation of 4-methylpiperazine rings.

Metabolites M16 (C_29_H_29_N_7_O_2_) and M23 (C_30_H_34_N_7_O), showing the molecular ions [M + H]^+^ at *m/z* 508.2454 and *m/z* 508.2817, were eluted at 10.75 min and 12.50 min, and they were 14 Da more than that of IM. For the prototype drug, M0, N-demethylated amide bond connecting to the benzene ring broke and formed *m/z* 203 fragment ion of M16. The fragment of *m/z* 203 further lost one CO and generated the fragment ion of *m/z* 175. The other fragment ions share the same metabolite pathway as the above. Therefore, the metabolite is considered to be the IM metabolite of carbonylation of N-methyl. The *m/z* 408 fragment ion of M23 was the M0 methylation followed by the removal of the 4-methyl-1-piperazinyl group, and it is presumed to be an N-methylation reaction on the amino group of the benzamide group or the amino group of 2-aniline on the pyrimidine ring. The fragment ion at *m/z* 264 was generated by the breakage of the amide bond after the breakage of the methyl group of C_4_ on the benzene ring of 2-anilinopyrimidine and the formation of a five-membered ring at this site with C_1_-N of the pyrimidine ring. Other fragment ions were also broken in the same way as above. Therefore, it was predicted that M23 is an amino group from the benzamide group of IM or an N-methylated metabolite from the amino group of 2-aniline on the pyrimidine ring.

Metabolite M15 (C_28_H_30_N_7_O), with a [M + H]^+^ of *m/z* 480.2505, was eluted at 10.52 min, which was 14 Da less than that of IM. M15 had the same fragment ion of *m/z* 394 as the above metabolites and two new fragments. One (*m/z* 119) formed via the separation of the carbonyl group from the benzene ring. The breakage of the amide bond to the benzene ring after N-demethylation of IM yields fragment ion of *m/z* 203, and the *m/z* 222 fragment ion was the methyl group of C_4_ from the benzene ring of 2-anilinopyrimidine after the breakage of this site and the C_1_-N of the pyrimidine ring to form a five-membered ring. C_1_, C_6_, C_4_, and C_5_ are broken and the intramolecular proton migration rearrangement was generated. The fragment ion at *m/z* 394 was broken in the same way as M6, so it is presumed that M15 was the N-demethylated metabolite of IM.

In addition, M11 (C_18_H_17_N_5_O), eluted at 9.50 min, gave a [M + H]^+^ at *m/z* 320.1498 (173 Da less than that of IM). The MS/MS spectra of M11 had a special peak at *m/z* 278, owing to the amide bond breaking (deacetylation), and the other *m/z* 261 was 17 Da lower than the fragment ion at *m/z* 278 of M11 as a result of losing NH_2,_ indicating that M11 is a metabolite of N-acetylation after amide bond hydrolysis of IM.

The [M + H]^+^ ion of M27 (C_24_H_21_N_5_O) at *m/z* 396.1818 was 97 Da lower than that of IM and eluted at 15.70 min. The product ion at *m/z* 378 was formed by losing a molecular of H_2_O via the amide bond; thus, M27 was generated by removal of the 4-methyl-1-piperazinyl from IM. In the MS/MS spectra, M26 (C_24_H_19_N_5_O_2_) showed the molecular ion [M + H]^+^ at *m/z* 410.1613 and was eluted at 15.70 min, which is 83 Da less than that of M0. The fragment ion at *m/z* 392 and the *m/z* 378 of M27 shared the same pathway of losing one molecular of H_2_O and *m/z* 261 fragment ions are generated via breakage between the N of the benzamide group and the benzene ring. Therefore, M26 may be a metabolite of formaldehyde generated from the methyl group at the C_4_ position of the benzene ring on the benzamide group of IM.

M17 (C_26_H_24_N_6_O) exhibited a protonated molecule ion [M + H]^+^ at *m/z* 437.2082 and was eluted at 11.09 min, which is 56 Da less than that of IM. Fracture mode was the same as IM. The fragment ion *m/z* 394 was formed via rupture of 4-methyl-1-piperazine group, indicating that 4-methyl-1-piperazinyl undergoes N-demethylation with simultaneous catalytic dehydrogenation. In addition, the loss of one molecule of methyl CH_3_ from the *m/z* 394 fragment ion resulted in the fragment ion of *m/z* 379, followed by *m/z* 290 through dehydrogenation of one molecule of the benzene ring. The fragment of *m/z* 160 was generated by the breakage of the amide bond after dehydrogenation of C_3_H_5_N, and it was predicted that M17 may be the dehydrogenation product of IM after dehydrogenation of one molecule of C_3_H_5_N from the metabolite.

M5 (C_30_H_32_N_6_O, eluted at 7.47 min) and M19 (C_29_H_30_N_7_O_3_, eluted at 11.54 min), showing the molecular ion [M + H]^+^ at *m/z* 492.2508 and 524.2397, were 1 Da different from that of IM. The metabolite lost hydrogen atom from carbonyl and N on phenylamino groups so that *m/z* 392 of M5 appeared in the MS/MS spectra and another one *m/z* 275 fragment ion is the product of breakage of N and benzene ring of benzamide group. As a result, M5 was presumed to be a metabolite of the dehydrogenation of N-4-methyl-3-[4-(3-pyridyl)-2-pyrimidine. The *m/z* 494 fragment ion produced by M19 was the prototype drug of IM. Therefore, it was considered to be the fragment ion of its broken substituted one-molecule hydroxyl and one-molecule carbonyl. The fragments of *m/z* 189, *m/z* 217, and *m/z* 394 were formed in the same way as M6 and M7. Based on the above results, it was concluded that M19 was the metabolite of IM 4-methyl-3-[4-(3-pyridyl)-2-pyrimidine by losing one molecule of hydroxyl and one molecule of carbonyl-substituted metabolite.

M12 (C_29_H_31_N_7_O_3_) and M22 (C_29_H_29_N_7_O_3_) exhibited a molecular ion at *m/z* 524.2409 and *m/z* 524.2396, which are 21 Da more than that of IM. In the MS/MS spectra of M12, *m/z* 506 fragment ion was the fragment ion generated from the substituted metabolite by removing H_2_O. Methyl pyridine ring (C_4_ of 2-aniline pyrimidine benzene ring) happens to break; then, the site on a pyrimidine ring of C_1_-N forms not only five-membered ring but also aminocarbonyl and N benzoyl break and, at the same time, produces *m/z* 290 and *m/z* 217. The structure of the 1-methylpiperazine group cracked the metabolite, resulting in the fragment ion m/z 99. As a result, M12 is molecular imatinib, a member of hydroxyl and carbonyl replacement metabolites (replacement occurred in 4-methyl-3-[4-(3-pyridyl)-2-pyrimidine structures). The fragment of *m/z* 290 was created via the breakage of the methyl group of C_4_ from the benzene ring of 2-anilinopyrimidine after the formation of a five-membered ring with C_1_-N of the pyrimidine ring and the break of the carbonyl group and N on the benzoylamino group, which also generates the *m/z* 217 fragment ion, and the *m/z* 99 fragment ion produced from the break of the 1-methyl piperazinyl group break generated presumably M12, that is, the 4-methyl-3-[4-(3-pyridyl)-2-pyrimidine of IM with one molecule of hydroxyl and one molecule of carbonyl-substituted metabolite. The *m/z* 478 fragment ion of M22 was generated by N-demethylation in the prototype of IM, and the *m/z* 247 fragment ion further deamidates the remaining carbonyl CO to produce the *m/z* 219 fragment ion of M22. Therefore, it was presumed to be oxidized to carboxylic acid on N-methyl, and M22 was a metabolite of oxidation of methyl to carboxylic acid on N-methyl of IM.

### 3.2. Metabolite Analysis in Bile

**Figure 6:**
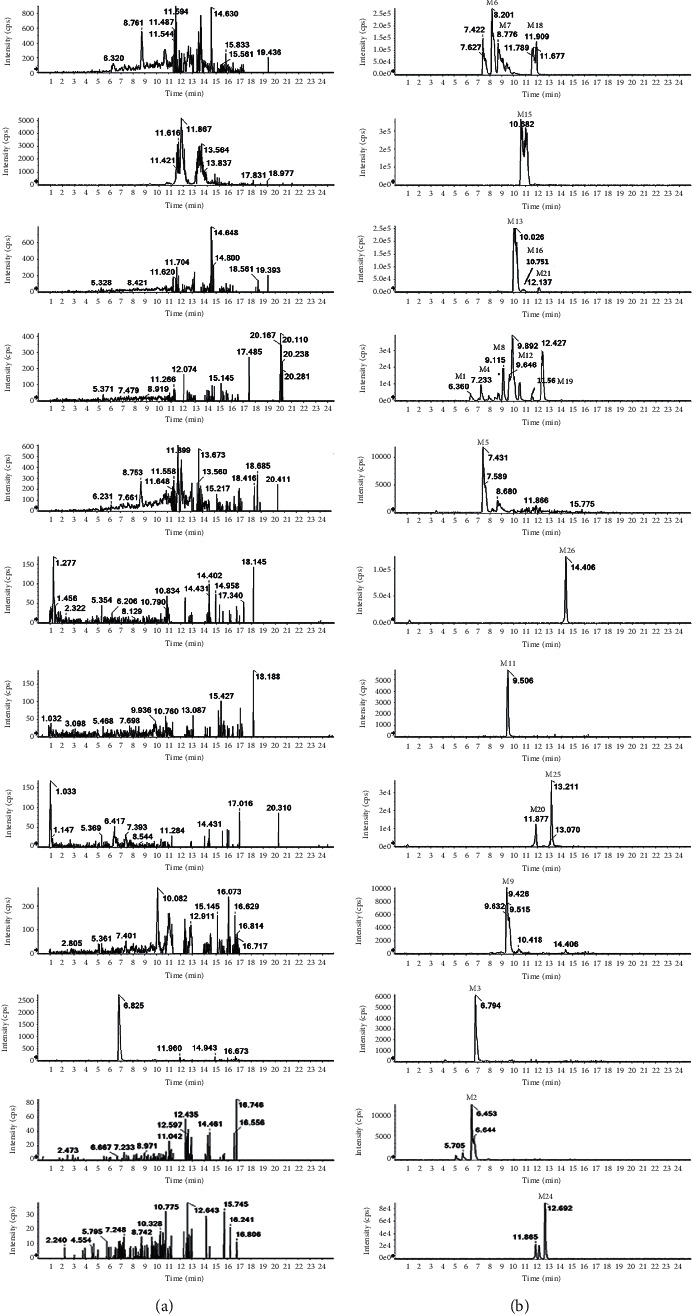
Extracted ion chromatograms (XIC) for metabolites in rats: (a) in blank bile sample; (b) in bile sample after an oral administration of IM.

Compared with the control group, 21 metabolites were detected in the bile. Moreover, 6 of them (M3, M9, M11, M17, M20, and M26) were newly discovered. The extracted ion chromatograms of all bile metabolites are shown in Figure 6. The chromatographic retention behavior of the metabolites, quasimolecular ions, and secondary fragment ions was comprehensively analyzed (Table 1). It was found that 12 of them were consistent with the metabolites in plasma, and the other 9 were different. Fragmental ions are shown in Figure 5.

M1, M4, and M8 shared the same formula (C_29_H_30_N_7_O_3_) with similar mass spectra and were eluted at the retention time of 9.10, 6.36, and 7.22 min. They showed molecule ions [M + H]^+^ of *m/z* 524.2412, 524.2414, and 524.2406, respectively. The loss of 30 Da can be detected in the MS/MS spectrum. It was speculated that they were the product of one molecule of hydroxyl substitution and one molecule of carbonyl substitution of IM.

M20 and M25 (C_24_H_20_N_5_O_3_) were eluted at 13.20 min and 11.83 min. They showed molecular ion [M + H]^+^ at *m/z* 426.1568 and 426.1582, which were 68 Da less than IM. The neutral loss of 18 Da (H_2_O) was obtained between the ions *m/z* 426 and 408. Fragment ion at *m/z* 261 was the product of the cleavage of N and the benzene ring on the benzamide. Therefore, it was speculated that the methyl group of 4-methylbenzamide was oxidized to a carboxylic acid. Based on the above results, it was inferred that M25 was a metabolite of IM depleted 4-methyl-1-piperazinyl carboxylic acid. The fragment ion at *m/z* 133 was generated by the CO-N cleavage of the amide bond of benzamide after M20 carbonyl substitution. The fragmentation of other ions was the same as the analysis. According to the metabolic law, it was speculated that the methyl group of 4-methylbenzamide was oxidized to aldehyde. Thus, it was inferred that M20 was formed by removal of N-[4-methyl-3-[4-(3-pyridyl) from 4-methyl-1-piperazinylbenzamide on IM-2-pyrimidinyl]amino]phenyl, one molecule of hydroxyl substitution, and 4-methyl benzamide part of the methyl oxidation to the metabolite of aldehyde.

M9 (C_24_H_23_N_6_O) was eluted at 9.41 min, and the [M + H]^+^ at *m/z* 411.1931 was 83 Da less than IM. The fragment ion at *m/z* 394 was obtained by the loss of NH_3_, and *m/z* 134 was the amide bond CO-N cleavage of benzamide after the loss of C_5_H_9_N. Therefore, it was an IM metabolite after losing 4-methyl-1-piperazinyl C_5_H_9_N.

M3 (C_15_H_22_N_3_O_2_), M2 (C_35_H_40_N_7_O_8_), and M24 (C_32_H_33_N_8_O_3_S) were phase II metabolites of IM, which are eluted at 6.78 min, 6.45 min, and 12.68 min, respectively. They showed molecular ion [M + H]^+^ at *m/z* 276.1708, 686.2936, and 609.2383. M3 was 318 Da less than IM. The product ion at *m/z* 258 implied the loss of H_2_O. The loss of 4-methyl-1-piperazinyl was obtained between *m/z* 258 and *m/z* 147. M3 was an N-acetylation metabolite after IM amide bond hydrolysis. M2 was 192 Da more than IM. The characteristic fragment ion at *m/z* 686 generated the ion at *m/z* 510 by the loss of glucuronic acid molecule (*m/z* 176). It was speculated that M2 may be the glucuronic acid conjugate after partial oxidation of N-4-methyl-3-[4-(3-pyridinyl)-2-pyrimidinyl]amino]phenyl of imatinib benzamide. M24 was 115 Da more than IM. The fragment ions at *m/z* 394 and *m/z* 215 are generated by the cleavage of benzamido benzyl and piperazine. The fragment ion at *m/z* 215 was composed of cysteine and 4-methyl-1-piperazine. M24 was speculated to be the metabolite of 4-methyl-1-piperazinyl with cysteine binding.

### 3.3. Metabolite Analysis in Urine

**Figure 7:**
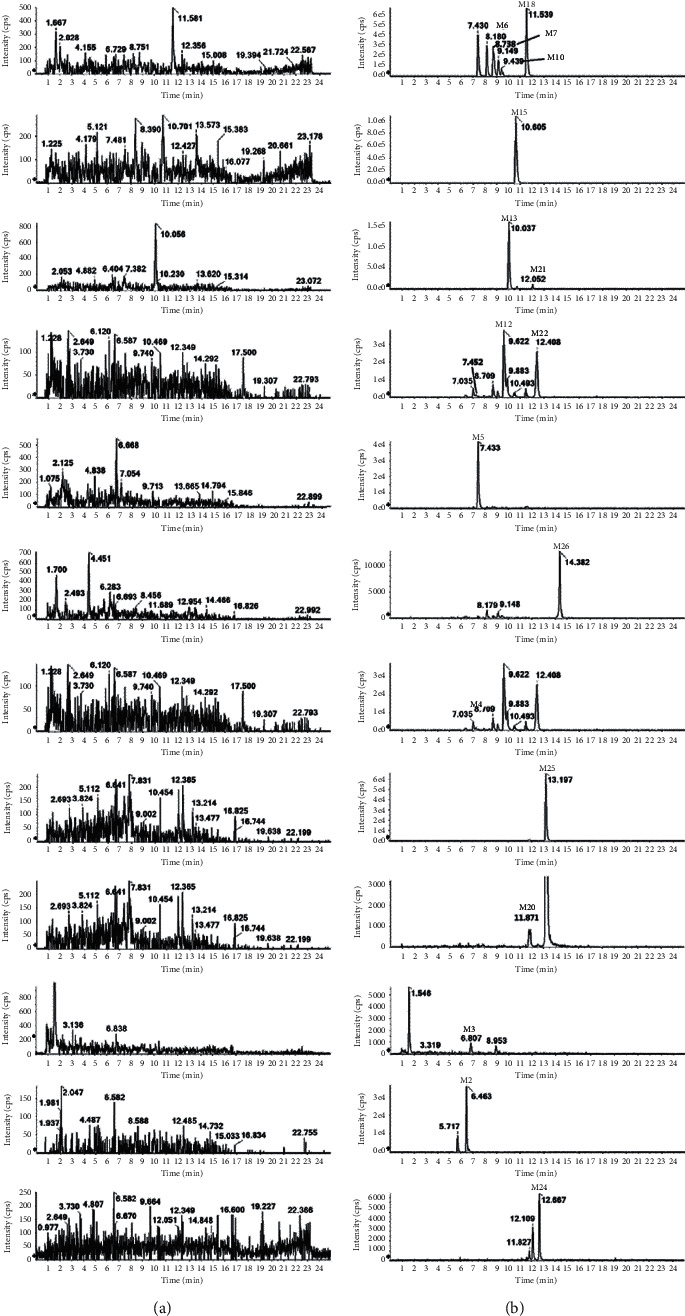
Extracted ion chromatograms (XIC) for metabolites in rats: (a) in blank urine sample; (b) in urine sample after an oral administration of IM.

Compared with blank urine samples, in addition to the IM prototype drug, 18 metabolites were detected. Ion chromatography of all urine metabolites extracted is shown in Figure 7. Comprehensive analysis of chromatographic retention behavior, quasimolecular ions, and secondary fragments ions are shown in Table 1.

### 3.4. Metabolite Analysis in Feces

**Figure 8:**
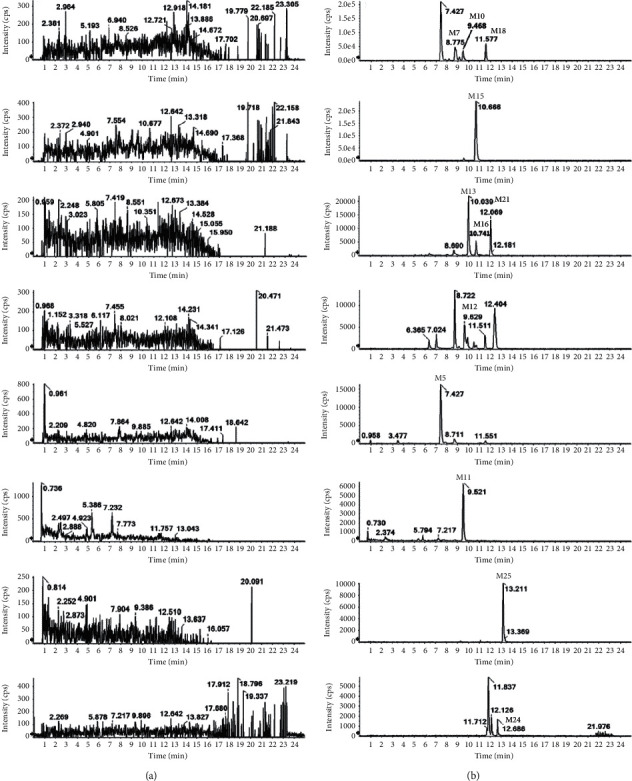
Extracted ion chromatograms (XIC) for metabolites in rats: (a) in blank feces sample; (b) in feces sample after an oral administration of IM.

Compared with blank stool samples, in addition to the IM prototype drug, 12 compounds were also detected. The extracted ion chromatograms of all feces metabolites are shown in Figure 8. Comprehensive analysis of chromatographic retention behavior, quasimolecular ions, and secondary fragments ions is presented in Table 1.

### 3.5. Metabolic Pathways of IM in Rats

**Figure 9:**
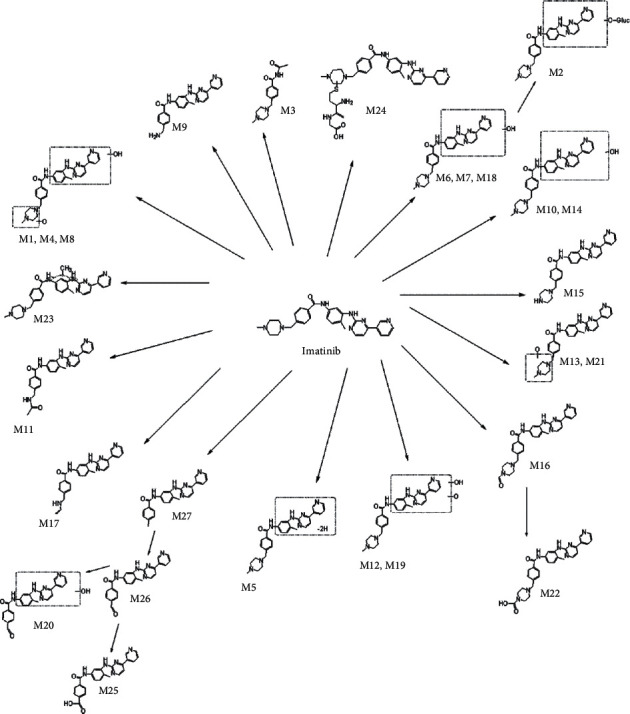
Proposed metabolic pathways of IM in rats.

In summary, the UHPLC-Q-TOF-MS/MS method was used to study the metabolism of IM in rats. A total of 27 metabolites were found, including 22 phase I metabolites and 5 phase II metabolites. The phase I metabolic pathways contained hydroxylation, oxidation, catalytic dehydrogenation, and dealkylation; the phase II metabolic pathways were N-acetylation, methylation, cysteine binding reactions, and glucuronidation. These metabolic pathways may also cross-link each other to generate secondary metabolites. Based on the above results, the main metabolic pathways of IM in rats are summarized in Figure 9.

## 4. Discussion

In the results of this experiment, M1-M2, M4-M8, M10, M12-M16, M18-M19, M21, and M24-M25 were previously reported metabolites of IM. Among them, the characteristic fragment ions and structures of M6, M7, M10, M14, M15, and M18 were consistent with those of M3, M8, M5, M6, N-demethyl metabolite, and M7 in the actual test results of Marull and Rochat [18]. In this experiment, the structures of M13, M21, and M25 were consistent with the previously reported structures of M29.6 and APG050 and M42.2 [23]. The oxides and dioxides in the results of Friedecký et al. (metabolites 59–61 and 65–74) may be consistent with M16 and M1, M4, M8, M12, and M19 in this study because they shared the same molecular formula and weight [20]. M5 was consistent with the previously reported dissociation products of M519 B because they had the same characteristic fragment ions and chemical structures [19]. M24 was also similar to the reported M609, and they all have fragment ions at *m/z* 215, *m/z* 394, *m/z* 478, and *m/z* 565 [24]. In addition, the experimental results of M2 may be the same as metabolite No. 25 of Rochat et al. [25], but they did not provide complete characteristic fragments ion and structural formula. Therefore, new content was added to this study. With regard to the effect of IM metabolites, N-demethylated metabolites of IM (M15 in this experiment) were the main metabolites of IM, although they had similar effects. However, some studies have shown that the pharmacological activity of N-demethyl IM was three times lower than that of IM [26]. Mlejnek et al. also found that N-demethyl IM had almost no therapeutic effect on CML through K562 cell experiments [17]. In addition, some studies have shown that some metabolic compounds, especially highly demethylated and sulfur-containing compounds (M15 and M24 in this experiment) may be related to IM adverse reactions [24, 26].

With regard to the newly discovered IM metabolites in this experiment, M22 is an IM metabolite with a carboxyl group, which was widely used in drug design and synthesis and was often used to form pharmacophore of various drugs. In addition, this group was reported to play a key role in the interaction between drugs and targets [27]. M3 and M11 were newly discovered N-acetylated metabolites in this experiment. Studies have shown that acetylated compounds can induce apoptosis in CML cells (k562) and IM-resistant CML cells (IR-K562). Therefore, these metabolites may have the potential of value for the drug development industry [28]. M23 was a newly discovered N-methylated metabolite. It has been reported that methylation biomarkers were closely related to drug resistance, and it may help with the study on drug resistance of CML [29]. M9 and M27 were the metabolites of dealkylation. Studies showed that the dealkylation metabolites of TKI had the same effect as the original drug. Therefore, M9 and M27, as newly discovered IM dealkylation metabolites, may also have the potential drug activity [21]. M26 and M20 were metabolites with aldehyde groups, which can lead to inactivation of cytochrome P450, drug interaction (DDI), and hepatotoxicity. They may provide a new way to investigate the toxicity of TKI drugs [16]. M17 was the first-phase metabolite of N-demethylation and catalytic dehydrogenation, and it was a new metabolite discovered for the first time.

The metabolites of IM and the fragment ions of IM were mainly produced by the cleavage of the C-N bond at the amide site, and the metabolic position of IM metabolites was determined by the increase or decrease of the molecular weight of the corresponding fragment ions. For example, N-oxidized metabolites tend to lose O on N during mass spectrometry cleavage, thus forming fragment ions lacking 16 Da. Therefore, the type and location of metabolic reaction can be determined by comparing the changes of the ion-to-nucleus ratio of fragments. In addition, in the analysis of IM and its phase II metabolites by two-phase full scan mass spectrometry, it was found that the glucuronic acid conjugate and cysteine conjugate first removed the binding group and obtained the corresponding mother nucleus.

## 5. Conclusions

In this study, the metabolic spectrum of IM in rats was studied using UHPLC-Q-TOF-MS/MS technique. 27 IM metabolites (18 known and 9 unknown) were found in plasma, bile, urine, and feces, including 18 in plasma, 21 in bile, 18 in urine, and 12 in feces. There were 22 metabolites in phase I. M6, M7, M10, M14, and M18 were formed by hydroxylation. M13, M16, M21, and M26 were formed by oxidation, and M5 was formed by catalytic dehydrogenation. M9, M15, and M27 were generated by dealkylation metabolism. M1, M4, M8, M12, M17, M19, M20, M22, and M25 were produced by cross-reaction. There were five phase II metabolites, M2 was formed by glucuronic acid-binding reaction, and M23 is through methylation. M11 and M3 were formed by N-acetylation and M24 was due to the cysteine binding reaction. IM and its phase I metabolites can be combined with endogenous substances to greatly improve the water solubility of metabolites and the level of drug excretion. However, the methylation metabolite M23 (II phase) of IM was on the contrary. Under the action of methylation, the methylated compound S-adenosyl methylthiamine (SAM) was used as a cofactor. After being catalyzed by methyltransferase, methyl was transferred to the O, S, N atoms of the substrate to form a less polar product, resulting in a decrease in the water solubility of the metabolite, making it difficult to be excreted. Since more metabolites were found in bile and urine, it was suggested that IM and its metabolites rely on bile and urine for metabolism and excretion.

## Figures and Tables

**Table 1:** Chromatographic retention times, precursor ions, and key fragments of IM and its metabolites in rat plasma, bile, urine, and feces sample.

No.	Formula of [M + H]^+^ ion	MS data	Error (ppm)	t_R_ (min)	MS/MS data	Plasma	Bile	Urine	Feces	No.	Formula of [M + H]^+^ ion
M1	C_29_H_30_N_7_O_3_^+^	524.2414	1.4	6.36	119,231,392,506	−	+	−	−	M1	C_29_H_30_N_7_O_3_^+^
M2	C_35_H_40_N_7_O_8_^+^	686.2936	0.4	6.45	217,410,510	−	+	+	−	M2	C_35_H_40_N_7_O_8_^+^
M3	C_15_H_22_N_3_O_2_^+^	276.1708	0.7	6.78	147,258	−	+	+	−	M3	C_15_H_22_N_3_O_2_^+^
M4	C_29_H_30_N_7_O_3_^+^	524.2406	−0.1	7.22	203,231,392,410,506	−	+	+	−	M4	C_29_H_30_N_7_O_3_^+^
M5	C_30_H_32_N_6_O^+^	492.2508	0.3	7.47	217,275,392	+	+	+	+	M5	C_30_H_32_N_6_O^+^
M6	C_29_H_32_N_7_O_2_^+^	510.2611	−0.2	8.19	111,217,189,277,410	+	+	+	−	M6	C_29_H_32_N_7_O_2_^+^
M7	C_29_H_32_N_7_O_2_^+^	510.261	−0.4	8.81	277,380,394,410	+	+	+	+	M7	C_29_H_32_N_7_O_2_^+^
M8	C_29_H_30_N_7_O_3_^+^	524.2412	1	9.1	119,203,231,410	−	+	−	−	M8	C_29_H_30_N_7_O_3_^+^
M9	C_24_H_23_N_6_O^+^	411.1931	0.8	9.41	134,247,264,394	−	+	−	−	M9	C_24_H_23_N_6_O^+^
M10	C_29_H_32_N_7_O_2_^+^	510.2611	−0.2	9.46	111,189,217,410	+	−	+	+	M10	C_29_H_32_N_7_O_2_^+^
M11	C_18_H_17_N_5_O^+^	320.1498	−2.5	9.5	261,278	+	+	−	+	M11	C_18_H_17_N_5_O^+^
M12	C_29_H_31_N_7_O_3_^+^	524.2409	0.6	9.76	99,217,290,506	+	+	+	+	M12	C_29_H_31_N_7_O_3_^+^
M13	C_29_H_30_N_7_O_2_^+^	508.2447	−1.6	10.08	203,222,231,247,394	+	+	+	+	M13	C_29_H_30_N_7_O_2_^+^
M14	C_29_H_32_N_7_O_2_^+^	510.261	−0.3	10.37	111,217,410	+	−	−	−	M14	C_29_H_32_N_7_O_2_^+^
M15	C_28_H_30_N_7_O^+^	480.2505	−0.2	10.52	119,203,222,394	+	+	+	+	M15	C_28_H_30_N_7_O^+^
M16	C_29_H_29_N_7_O_2_^+^	508.2454	−0.3	10.75	175,203,222,231,247,000	+	+	−	+	M16	C_29_H_29_N_7_O_2_^+^
M17	C_26_H_24_N_6_O^+^	437.2082	−0.6	11.09	160,290,379,394	+	−	−	−	M17	C_26_H_24_N_6_O^+^
M18	C_29_H_32_N_7_O_2_^+^	510.2598	−2.9	11.54	189,277,394	+	+	+	+	M18	C_29_H_32_N_7_O_2_^+^
M19	C_29_H_30_N_7_O_3_^+^	524.2397	−1.8	11.54	189,217,394,494	+	+	−	−	M19	C_29_H_30_N_7_O_3_^+^
M20	C_24_H_20_N_5_O_3_^+^	426.1582	4.9	11.83	133,277,408	−	+	+	−	M20	C_24_H_20_N_5_O_3_^+^
M21	C_29_H_30_N_7_O_2_^+^	508.2443	−2.5	12.06	222,231,247,394	+	+	+	+	M21	C_29_H_30_N_7_O_2_^+^
M22	C_29_H_29_N_7_O_3_^+^	524.2396	−2.1	12.42	219,247,394,478	+	−	+	−	M22	C_29_H_29_N_7_O_3_^+^
M23	C_30_H_34_N_7_O^+^	508.2817	−0.5	12.5	119,189,217,264,408	+	−	+	−	M23	C_30_H_34_N_7_O^+^
M24	C_32_H_33_N_8_O_3_S^+^	609.2383	−1.3	12.68	171,215,394,478,565	−	+	+	+	M24	C_32_H_33_N_8_O_3_S^+^
M25	C_24_H_20_N_5_O_3_^+^	426.1568	1.7	13.2	149,261,408	−	+	+	+	M25	C_24_H_20_N_5_O_3_^+^
M26	C_24_H_19_N_5_O_2_^+^	410.1613	0.4	14.17	133,261,392	+	+	+	−	M26	C_24_H_19_N_5_O_2_^+^
M27	C_24_H_21_N_5_O^+^	396.1818	−0.6	15.7	119,378	+	−	−	−	M27	C_24_H_21_N_5_O^+^

## Data Availability

All data generated or analyzed during this study are included within this article.
